# Plasma short-chain fatty acids are associated with COPD and COPD phenotypes

**DOI:** 10.1186/s12890-026-04348-2

**Published:** 2026-05-16

**Authors:** Anders Ørskov Rotevatn, Tomas Mikal Eagan, Adrian McCann, Johnny Laupsa-Borge, Solveig Tangedal, Gunnar Reksten Husebø, Rune Nielsen

**Affiliations:** 1 Department of Clinical Science, Faculty of Medicine, University of Bergen, Bergen, Norway; 2 Department of Thoracic Medicine, Haukeland University Hospital, Post Office Box 1400, Bergen, N-5021 Norway; 3 Bevital AS, Bergen, Norway

**Keywords:** COPD Chronic obstructive pulmonary disease, Metabolome, Formate, SCFA Short-chain fatty acids, Propionate, Isobutyrate

## Abstract

**Background and aim:**

Short-chain fatty acids (SCFAs) are metabolites largely produced by the gut microbiota, except for formate, which is also produced by the host. SCFAs may influence COPD development by modulating the immune system. This study examined the relationships between circulating SCFA concentrations and COPD and COPD phenotypes.

**Methods:**

We report findings from a single-centre observational study with 130 patients with COPD and 103 controls. Eight SCFAs (formate, acetate, propionate, butyrate, isobutyrate, valerate, isovalerate and α-methylbutyrate) were quantified in plasma using isotope-labelled gas chromatography-tandem mass spectrometry. Associations between SCFAs, COPD status, and COPD phenotypes (severely obstructive COPD, emphysema, eosinophilic COPD, frequent exacerbations, and symptom burden) were evaluated using multivariable regression analyses adjusted for age, sex, smoking, and body composition.

**Results:**

Plasma formate concentrations were inversely associated with COPD status (adjusted OR 0.33; 95% CI 0.15 to 0.66; *p* = 0.004). Propionate and isobutyrate concentrations were inversely associated with severely obstructive COPD (adjusted ORs 0.46; 95% CI 0.24 to 0.86; *p* = 0.018, and 0.33; 95% CI 0.14 to 0.75; *p* = 0.012, respectively). Propionate concentrations were also inversely associated with frequent exacerbations of COPD (adjusted OR 0.23; 95% CI 0.07 to 0.62; *p* = 0.007). Isovalerate was positively associated with symptom burden (CAT score) (estimate 1.76; 95% CI 0.20 to 3.32; *p* = 0.027). No consistent associations were observed for emphysema or eosinophilic COPD.

**Conclusions:**

SCFA formate was inversely related to COPD. Several SCFAs were associated with specific COPD phenotypes. Future studies should investigate the underlying mechanisms linking SCFAs to COPD and include the often-overlooked formate, while acknowledging formate’s dual origin.

## Introduction

Chronic obstructive pulmonary disease (COPD) is a progressive lung disease characterised by emphysema and chronic inflammation in the airways. The clinical presentation is heterogeneous, with patients differing in degree of airflow impairment, symptom burden, markers of type 2 inflammation (e.g., blood eosinophil count), exacerbation frequency, and extent of emphysema. COPD develops in response to repeated injury from inhaled toxic gases, such as cigarette smoke. However, not all smokers develop COPD. What drives inflammation, and to varying degrees in different patients, is not fully understood. The immune response resembles a chronic infection, and microbial dysbiosis is a possible factor. Studies have shown a dysbiosis in the airway microbiota in patients with COPD [1, 2], but whether it is a cause or an effect remains unknown.

The gut microbiota, the body’s largest microbial community, may also be relevant in chronic lung disease [3]. Differences in the gut microbial composition have been observed between individuals with COPD or emphysema and healthy controls [4–8]. Although the clinical relevance of these findings is still unclear, faecal transplants from patients with COPD have induced lung inflammation and COPD-like changes in mice [6]. The underlying mechanisms are not well understood, but short-chain fatty acids (SCFAs) have been proposed as possible mediators.

SCFAs are gut-derived fatty acids with 1–6 carbon atoms, primarily produced by bacterial fermentation of undigested dietary fibre. However, branched SCFAs (such as isobutyrate, isovalerate and α-methylbutyrate) mainly arise from bacterial fermentation of branched amino acids. Although some SCFAs (acetate, propionate and butyrate) can be produced endogenously in negligible amounts, only formate is produced in substantial amounts by the host. Therefore, except for formate, circulating SCFA concentrations predominantly reflect gut microbial activity. Gut microbiota-derived SCFAs are detectable in the lungs [9], and both alveolar macrophages and alveolar type 2 cells express SCFA receptors [9]. Microbial metabolites, such as propionate and lipopolysaccharides, can influence alveolar immune tone and macrophage metabolism [9].

Only two small studies have previously compared SCFA concentrations in plasma between patients with COPD and controls, with conflicting results. One reported reduced acetate concentrations in COPD [10]. In the other, COPD smokers were compared with non-COPD smokers without finding differences, but the number of participants (6 per group) was likely too low to detect biological differences [11]. Others have reported lower acetate in feces of patients with COPD [6] and emphysema [12]. Additionally, faecal isobutyrate and isovalerate were reported to be lower in patients with severely obstructive COPD [6].

The MicroCOPD study included 130 patients with COPD and 103 controls with clinical data on exacerbation frequency, blood eosinophil level, lung function, symptom score, and emphysema. The current study aimed to investigate whether plasma SCFA concentrations differed between patients with COPD and controls, and according to COPD phenotypes.

## Methods

### Study design and population

The MicroCOPD study is a single-centre observational study (2012–2015, Haukeland University Hospital, Norway). The main objective was to investigate the role of microbiota in COPD, using bronchoscopy to sample the lower airways. Eligible participants were ≥ 40 years of age and included never, former, and current smokers. Inclusion was postponed if subjects had received antibiotics or oral corticosteroids within the preceding two weeks, or if they had symptoms of an ongoing COPD exacerbation. COPD diagnosis and control status were confirmed by experienced pulmonologists based on medical history and post-bronchodilator spirometry. Exclusion criteria were high bleeding risk, cardiac valve prosthesis, acute coronary syndrome within 6 weeks, cancer within the past five years, or significant immunosuppression. The controls had no history of lung or airway disease. A detailed protocol for the MicroCOPD study has previously been published [13].

### Measurements

At inclusion, participants underwent blood sampling and structured interviews, including smoking history, symptom burden (COPD Assessment Test; CAT), and time since last antibiotic treatment. Spirometry was performed using a Viasys Vmax ENCORE 30 min after inhalation of 400 µg of salbutamol administered through a large-volume spacer. Body composition was measured by bioelectrical impedance. Cachexia was defined as having a fat-free mass index (kg/m^2^) of < 14 for women and < 17 for men, while obesity was defined as a fat mass index (kg/m^2^) of > 13.5 in women and > 9.3 in men [14]. Thoracic computed tomography (CT thorax) scans were performed using a Siemens Somatom Definition Flash scanner. Emphysema was defined as > 10% low attenuating area (LAA) at density threshold −950 Hounsfield units (HU), using the free 3D Slicer software [15]. Blood counts were measured using standard laboratory methods. The plasma samples were collected according to standard phlebotomy techniques into EDTA-vacutainer tubes. Plasma separation was performed by centrifugation at 2,150 g for 15 min at 4 °C, after which the plasma was aliquoted and stored at −80 °C until analysis.

### Targeted analyses of SCFAs

Eight SCFAs – formate, acetate, propionate, butyrate, isobutyrate, valerate, isovalerate and α-methylbutyrate – were targeted for quantification in plasma using isotope-labelled internal standards for each metabolite and gas chromatography–tandem mass spectrometry (GC–MS/MS) at Bevital AS, Bergen, Norway (https://bevital.no/). The limits of detection for the metabolites were as follows: formate, 7.0 µmol/L; acetate, 5.0 µmol/L; propionate, 0.06 µmol/L; butyrate, 0.2 µmol/L; isobutyrate, 0.05 µmol/L; valerate, 0.05 µmol/L; isovalerate, 0.02 µmol/L; and α-methylbutyrate, 0.05 µmol/L. Within- and between-day coefficient of variation ranged from 3 to 7% and 3–8%, respectively.

In addition, we also calculated the total sum of SCFAs and the ratio of total to branched SCFAs (isobutyrate, isovalerate and α-methylbutyrate), the latter serving as an inverse marker of the degree of protein fermentation.

### Statistical analysis

Statistical analyses were conducted in R (version 4.4.1). Continuous data are presented as mean ± SD, and categorical variables as n (%) unless otherwise specified. Left-censored missing values of SCFA concentrations due to lower than the limit of detection or quantification were considered missing not at random and imputed using the GSimp procedure [16].

Associations between COPD and SCFA concentrations were assessed using unconditional logistic regression with COPD (yes/no) as the binary outcome: Three models were fitted: (i) unadjusted, (ii) adjusted for age and sex, and (iii) adjusted for age, sex, smoking, and body composition. Due to their multiplicative nature, SCFA concentrations were log_2_-transformed. With this transformation, the odds ratio reflects the change in odds associated with a doubling in SCFA concentrations.

Among participants with COPD, associations between COPD phenotypes and log_2_-transformed SCFA concentrations were investigated similarly using logistic regression with the following binary outcomes: emphysema vs. no emphysema, severely obstructive COPD as defined as forced expiratory volume in 1 s (FEV_1_) < 50% vs. FEV_1_ ≥50% of predicted values, high vs. low eosinophils (≥/<0.3 × 10^9^/L), and frequent vs. infrequent exacerbations (≥ 2/0–1 exacerbations of COPD the preceding year). For frequent exacerbations, adjustments were limited to age, sex, and smoking. Associations between symptom burden and SCFA concentrations were analysed using linear regression. Models were fitted (i) unadjusted, (ii) adjusted for age and sex, and (iii) additionally adjusted for smoking and body composition. With log2-transformation, the regression estimates the expected change in CAT score associated with a doubling of SCFA concentrations.

As a complementary analysis aimed at estimating group differences in SCFA concentrations, we fitted generalised least squares (GLS) models allowing for unequal residual variances between groups. Group was the main predictor, comparing COPD vs. controls, and each binary COPD phenotype (emphysema vs. no emphysema, severe airflow obstruction vs. non-severe, high vs. low blood eosinophils, and frequent vs. infrequent exacerbations). Because most biochemical variables fit a log-normal (multiplicative) distribution equally well or better than a normal (additive) distribution [17], SCFA values were transformed using the natural logarithm (ln). Relative between-group differences are expressed as sympercents (s%), which are additive and symmetric percentage differences on the 100 ln scale [18]. This relative measure is calculated as the difference between the natural logs of two numbers multiplied by 100. GLS was also used to evaluate the associations between symptom burden (CAT score) and natural log-transformed SCFA concentrations, with CAT score included as a continuous predictor. In this context, the relative measure represents the symmetric percentage change (s%) in SCFA concentration per 1-point increase in CAT score. All GLS models were fitted (i) unadjusted, (ii) adjusted for age and sex, and (iii) additionally adjusted for smoking and body composition.

Because analyses of SCFAs in relation to COPD status and phenotypes were explicitly exploratory and hypothesis-generating, we did not apply formal adjustments for multiple testing. A nominal two-sided alpha of 0.05 was used, and we report exact p-values with effect estimates and 95% confidence intervals to allow readers to judge the strength and precision of the evidence. All findings should be interpreted as provisional and in need of confirmation in independent cohorts.

## Results

Participant demographics and clinical characteristics are summarised in Table 1. Compared with controls, patients with COPD had greater smoking exposure, reduced lung function, and a higher prevalence of cachexia. In the COPD group, 24% had emphysema, 39% had FEV_1_ <50%, 35% had eosinophilic COPD, and 13% reported frequent COPD exacerbations.

**Table 1:** The demographic and clinical characteristics of the study participants

Characteristic	Control	COPD	Overall
	*N* = 103	*N* = 130	*N* = 233
Age	65.25 (± 8.64)	67.22 (± 7.35)	66.35 (± 7.99)
Sex (Male)	60 (58%)	76 (58%)	136 (58%)
Smoking status			
Never	19 (18%)	0 (0%)	19 (8.2%)
Former	59 (57%)	100 (77%)	159 (68%)
Current	25 (24%)	30 (23%)	55 (24%)
Body composition			
Normal	78 (76%)	81 (62%)	159 (69%)
Cachexia	10 (9.8%)	27 (21%)	37 (16%)
Obese	14 (14%)	22 (17%)	36 (16%)
Missing	1	0	1
FVC % of predicted	112 (± 13)	94 (± 18)	102 (± 18)
FEV_1_% of predicted	104 (± 12)	57 (± 19)	78 (± 29)
FEV_1_/FVC-ratio	0.73 (± 0.05)	0.47 (± 0.12)	0.59 (± 0.17)
Eosinophilic COPD (Blood eosinophils ≥ 0.3 × 10^9^)		46 (35%)	
Emphysema (≥ 10% LAA at -950 HU threshold)		28 (24%)	
Missing		14	
FEV_1_ <50%		51 (39%)	
Frequent COPD exacerbations (≥ 2 COPD exacerbations previous 12 months)		17 (13%)	
Missing		1	
Symptom burden (CAT score)		16 (± 8)	
Missing		2	

Data are presented as n (%) or mean ± SD

*BMI* body mass index, *LAA* low attenuating area, *HU* Hounsfield units, *FEV1* forced expiratory volume in 1 s, *FVC* forced vital capacity, *CAT *COPD Assessment Test

### COPD vs. controls

The geometric mean (SD range) of the plasma SCFA concentrations in controls and patients with COPD is presented in Table 2. Plasma acetate concentrations were highest, followed by formate. Valerate had the lowest overall plasma concentrations and the highest number of left-censored data (missing not at random) at 111 (47.6%), with butyrate second highest at 8 (3.4%) left-censored data.

**Table 2:** Concentrations of plasma SCFAs in COPD and controls

SCFAs, µmol/L	Control	COPD	*p*-value^2^
	*N* = 103	*N* = 130	
Formate	13.0 (10.5–16.1)	11.3 (7.35–17.5) ^1a^	**< 0.001**
Acetate	35.78 (20.56–62.25)	35.49 (16.06–78.45)	0.644
Propionate	0.95 (0.65–1.39)	0.99 (0.64–1.53)	0.282
Butyrate	0.44 (0.20–0.98)^1b^	0.45 (0.23–0.87)^1c^	0.732
Isobutyrate	0.25 (0.19–0.34)	0.26 (0.19–0.37)	0.334
Valerate	0.05 (0.03–0.10)^1d^	0.06 (0.03–0.11)^1e^	0.908
Isovalerate	0.32 (0.17–0.62)	0.30 (0.16–0.57)	0.418
α-methylbutyrate	0.12 (0.09–0.17)	0.12 (0.08–0.18)^1f^	0.792
Total SCFAs	51.66 (33.07–80.72)	51.02 (25.93–100.39)	0.752
Ratio of total to branched SCFAs	70.59 (44.37–112.30)	71.80 (35.10–146.89)	0.560

Geometric mean (SD range). ^1^n (%) missing not at random, n (%) values imputed groupwise by the GSimp method; ^1a^ 6 (4,6%), ^1b^ 3 (2,9%), ^1c^ 5 (3,8%), ^1d^ 46 (44,7%), ^1e^ 65 (50,0%), ^1f^ 1 (0,8%). ^2^P-values from linear regression modeling (generalised least squares), including a variance function structure allowing for different variances across groups and corrected for age, sex, smoking status and body composition

**Fig. 1:**
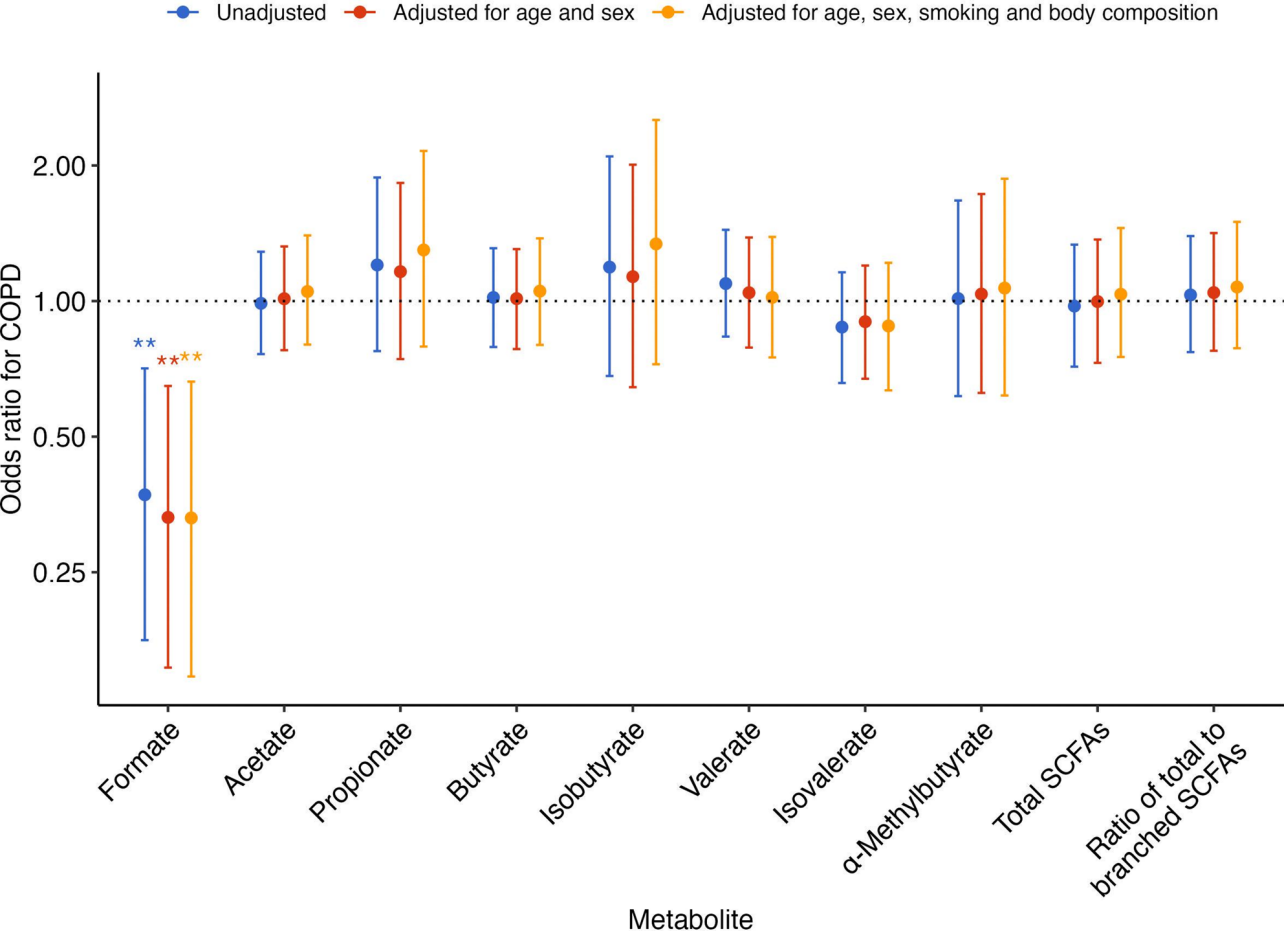
Associations between log_2_-transformed metabolite concentrations and COPD status (COPD vs. controls) from logistic regression analyses. Odds ratios represent the multiplicative change in odds associated with a doubling of metabolite concentrations. ** *p* < 0.01

**Fig. 2:**
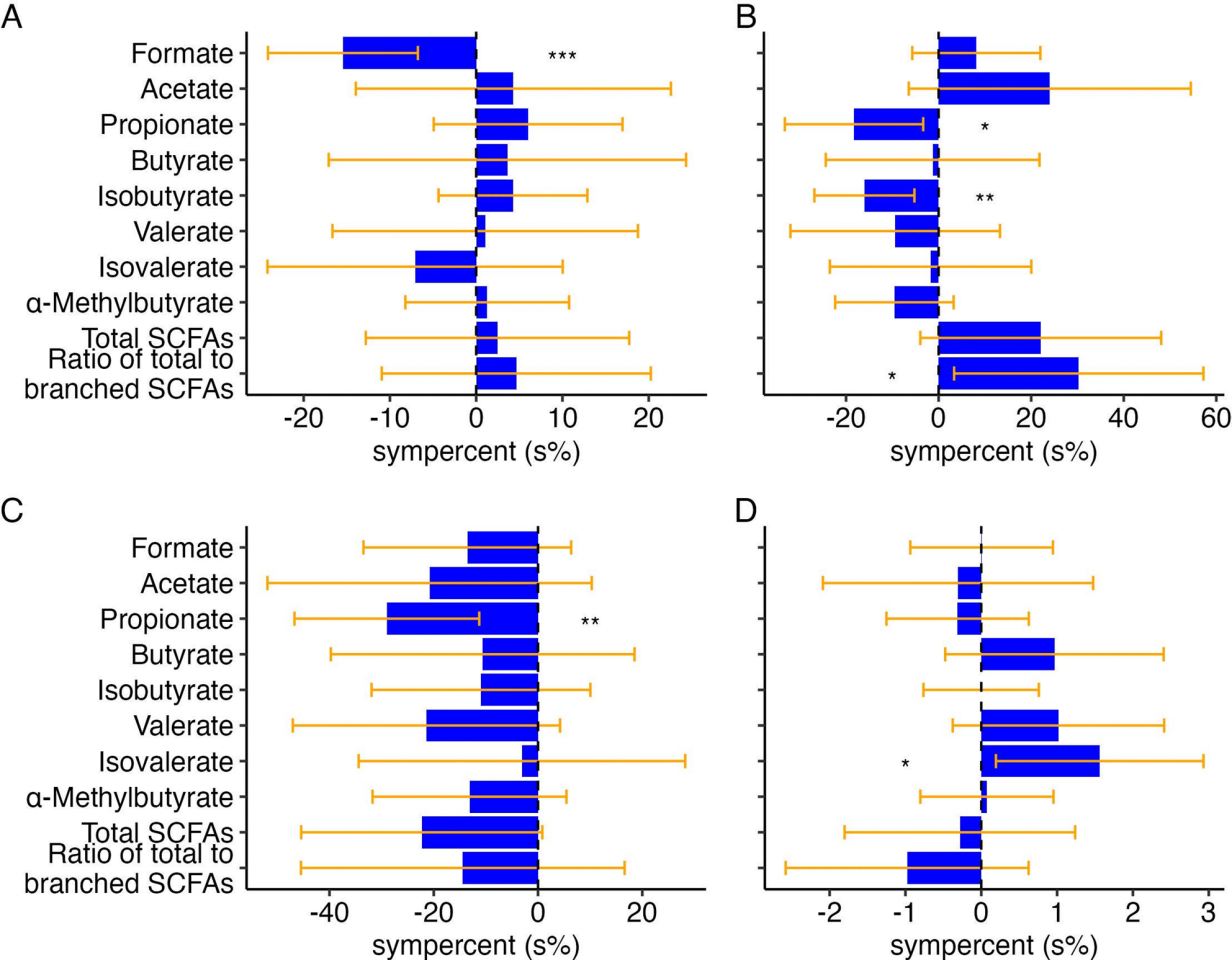
Group differences in SCFA concentrations expressed as sympercents (s%) with 95% confidence intervals according to **A** COPD status (COPD vs. controls), and among participants with COPD with: **B** FEV1 < 50%, **C** frequent exacerbations and **D** symptom score, all adjusted for age, sex, smoking status and body composition. * *p* < 0.05. ** *p* < 0.01. *** *p* < 0.001

A doubling in plasma formate concentrations was associated with 67% lower odds of COPD (adjusted OR 0.33; 95% CI 0.15 to 0.66; *p* = 0.004; Fig. 1). None of the other SCFAs were significantly associated with COPD status (all *p* > 0.05). Consistent with these findings, formate concentrations were lower in patients with COPD than in controls, with an adjusted difference of −15.5 s% (95% CI −24.1 to −6.8; *p* < 0.001) after adjustment for age, sex, smoking status and body composition (Fig. 2).

### Severely obstructive COPD

**Fig. 3:**
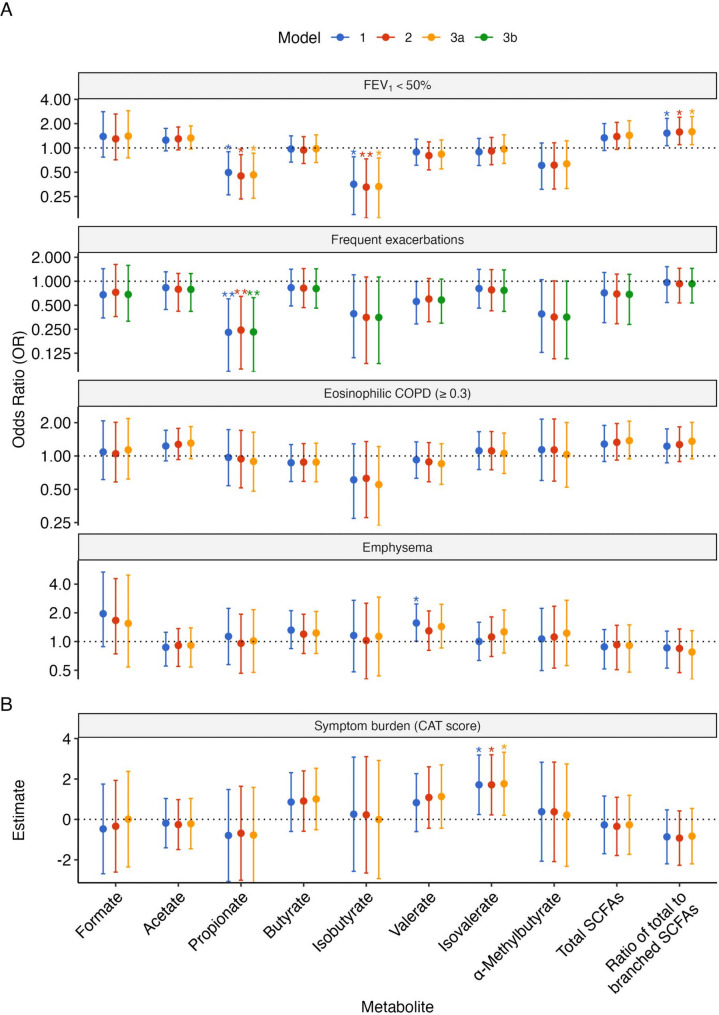
Associations between log_2_-transformed metabolite concentrations and COPD phenotypes. Panel **A** shows associations with dichotomic phenotypes, and Panel **B** shows associations with symptom score, numeric). Model 1 is unadjusted. Model 2 is adjusted for age and sex. Model 3a is adjusted for age, sex, smoking status and body composition. Model 3b is adjusted for age, sex and smoking. Because metabolite concentrations are log_2_-transformed, odds ratios from logistic regression represent the multiplicative change in odds associated with a doubling of metabolite concentration, whereas regression coefficients from linear regression represent the change in symptom score per doubling of metabolite concentration. * *p* < 0.05. ** *p* < 0.01

FEV_1_ <50% of predicted was associated with lower plasma propionate (adjusted OR 0.46; 95% CI 0.24 to 0.86; *p* = 0.018) and isobutyrate (adjusted OR 0.33; 95% CI 0.14 to 0.75; *p* = 0.012). The ratio of total to branched SCFAs was positively associated with severely obstructive COPD (adjusted OR 1.59; 95% CI 1.10 to 2.45; *p* = 0.021) (Fig. 3).

Consistently, propionate and isobutyrate concentrations were lower in those with FEV_1_ <50% (−18.28 s%; 95% CI −33.23 to −3.33 s%; *p* = 0.018 and −16.04 s%; 95% CI −26.85 to −5.23 s%; *p* = 0.004), while the ratio of total to branched SCFAs was greater (30.28 s%; 95% CI 3.35 to 57.22 s%; *p* = 0.029). All estimates were adjusted for age, sex, smoking status and body composition (Fig. 2).

### Frequent exacerbators

Plasma propionate concentration was inversely associated with frequent exacerbations of COPD (adjusted OR 0.23; 95% CI 0.07 to 0.62; *p* = 0.007). Valerate and α-methylbutyrate did not reach the predefined significance threshold, but they showed a trend towards inverse associations with frequent exacerbations (adjusted *p* = 0.093 and 0.068, respectively).

Consistently, propionate concentrations were lower in patients with COPD with frequent exacerbations (−29.00 s%; 95% CI −46.72 to −11.27; *p* = 0.002; adjusted for age, sex, smoking and body composition). Valerate and α-methylbutyrate concentrations were also lower in frequent exacerbators in unadjusted analyses (−33.11 s%; 95% −58.01 to −8.21; *p* = 0.010 and −18.21 s%; 95% CI −35.67 to −0.76; *p* = 0.043). When adjusting for age and sex, the reduction remained significant (−27.59 s%; 95% CI −52.54 to −2.64; *p* = 0.032 and −18.87; 95% CI −38.90 to −2.68; *p* = 0.037, respectively). However, these associations were no longer significant after additional adjustment for smoking status and body composition (*p* = 0.104 and 0.169, respectively).

### Eosinophilic COPD

No SCFAs were significantly associated with eosinophilic COPD (*p* > 0.05), and there were no significant differences in SCFA concentrations between patients with high and low blood eosinophils (*p* > 0.05).

### Emphysema

Valerate was barely significantly associated with emphysema according to unadjusted analysis (OR 1.56; 95% CI 1.01 to 2.48; *p* = 0.049) but was not significant in the adjusted models (Fig. 3). None of the other SCFAs were significantly associated with emphysema among patients with COPD (*p* > 0.05).

### Symptom burden

A doubling of isovalerate concentrations was associated with a multiplicative increase in CAT score of 1.76 (95% CI 0.20 to 3.32; *p* = 0.027), adjusted for age, sex, smoking status and body composition. Consistently, isovalerate concentrations increased by 1.56 s% per one-point increase in CAT score (95% CI 0.19 to 2.93; *p* = 0.027; adjusted for age, sex, smoking and body composition).

## Discussion

In this study, we investigated the associations between plasma SCFA concentrations in patients with COPD compared with controls, as well as in COPD patients with different disease manifestations. Overall, formate concentrations were lower in patients with COPD. Specific SCFAs were related to distinct COPD phenotypes. Propionate was lower in those with FEV_1_ <50% and in patients with frequent exacerbations, isobutyrate was lower in patients with severely obstructive COPD, and isovalerate was positively associated with symptom burden.

### Formate and COPD status

Formate concentrations were the second highest among the SCFAs in our study participants, with concentrations more than tenfold greater than the third highest. While formate has not been included in previous SCFA-focused studies on patients with COPD [6, 10–12], untargeted metabolomic approaches using nuclear magnetic resonance (NMR) for metabolome analysis have reported altered formate levels in COPD. One study analysing exhaled breath condensate (EBC) observed lower formate concentrations in patients with COPD compared with controls [19]. Another study reported that serum formate concentrations and lung function increased in patients with COPD after three months of doxycycline treatment, but not in controls [20], and a third study demonstrated a positive association between urinary formate and lung function, but not with plasma formate, possibly due to the limited sensitivity of their analytical methods [21]. Additionally, serum formate was found to be lower in patients with acute respiratory failure than those with stable COPD [22].

In contrast, Zheng et al. found higher serum formate in 54 patients with COPD compared with 74 controls [23], but they used untargeted NMR for quantification, which is less sensitive and specific than the targeted GC–MS/MS methods we applied. Airoldi et al. reported higher formate in EBC from alpha_1_-antitrypsin-deficient patients with emphysema compared with healthy controls [24], but the study included only 22 participants. Circulating formate has also been found to be reduced in other maladies such as HER2 + breast cancer, non-small cell lung cancer and obesity [25].

Formate is an intermediate metabolite in the one-carbon metabolism [26], produced by both host mitochondrial pathways (primarily serine and glycine catabolism via SHMT2 and MTHFD2) and by anaerobic bacterial fermentation in the gut. Circulating formate therefore reflects the host’s metabolic state, mitochondrial one-carbon flux, and microbiome activity. As a key one-carbon donor, formate supports de novo purine and pyrimidine synthesis, histone and protein methylation, and immune cell proliferation. Its dual origin complicates interpretation, but the inverse association observed here suggests that reduced circulating formate in COPD reflects broader metabolic stress —specifically impaired mitochondrial one-carbon metabolism and systemic energy deficits — rather than gut-derived immunomodulation alone. These findings highlight the need for further investigation of formate as a potential marker of COPD-related metabolic dysfunction.

### Propionate

Propionate concentrations were lower in those with FEV_1_ <50% and in frequent exacerbations, somewhat consistent with Otake’s study, which reported a positive correlation between propionate and FEV_1_/FVC [11], although Li et al. found no significant difference in propionate according to severity of COPD [6]. Propionate has previously been reported to be increased in EBC from patients with COPD [19] and alpha_1_-antitrypsin-deficient patients with emphysema [24] compared with controls. In mice, propionate enhances haematopoiesis of dendritic cell precursors and leads to impaired activation of TH2 effector cells in the lung and resolution of allergic airway inflammation. These effects depended on G protein–coupled receptor 41 (Free fatty acid receptor 3) [27]. Propionate also represses IL-17 by human γδ T cells [28]. However, we found no significant association between propionate (or any other SCFAs) and eosinophilic COPD.

### Isobutyrate and isovalerate

Isobutyrate was also lower in patients with severely obstructive COPD, consistent with an earlier study focusing on protein degradation in COPD [29]. Isovalerate was positively associated with symptom burden, a previously unreported finding. Branched SCFAs such as isobutyrate and isovalerate are understudied, although high colonic and systemic concentrations have been linked to reduced gut and metabolic health [30].

### Acetate

Acetate had the highest concentrations of the measured SCFAs in our material. We found no differences in acetate between COPD and controls, consistent with Otake et al. [11]. Other studies have reported lower serum [10] and faecal [6, 12] acetate in patients with COPD and emphysema. Otake et al. also reported reduced acetate and propionate in current smokers compared with non-smokers [11]. We adjusted for smoking in our analysis, but this did not significantly affect associations between COPD, COPD phenotypes and SCFAs.

### Emphysema

Contrary to expectations, we found no strong associations between emphysema and SCFAs. A weak positive correlation with valerate was no longer significant after adjusting for age and sex. Valerate had the lowest concentrations among the SCFAs, with many missing values imputed, which limits the reliability of our observations. Our results differ from those of Lee et al., who reported lower faecal acetate in patients with emphysema [12]. There is a considerable concentration gradient between colonic and systemic SCFA levels [30], which could allow for different patterns in serum and faecal samples. Additionally, while emphysema usually slowly evolves, reflecting chronic pathological processes, we measured SCFAs only at a single time point, and cannot exclude temporal variation in SCFA concentrations. Lee et al. reported therapeutic effects of acetate and propionate in a smoking-induced murine model of emphysema [12]. This finding aligns with Kim et al., who found that antibiotics aggravated emphysema development and changed the microbiota, whereas subsequent SCFA administration attenuated this effect [31]. Other murine studies have shown that high-fibre diets increase SCFA and attenuate emphysema [32, 33]. Large human studies similarly associate higher dietary fibre with lower COPD risk [34–36] and higher lung function [37]. Long-term dietary intervention trials should further explore whether systemic SCFA changes mediate protective effects. Additionally, future studies on COPD and SCFAs should include formate.

### Strengths and limitations

Strengths include detailed clinical characteristics of participants, use of highly sensitive isotope-labelled GC–MS/MS, and measurement of circulating SCFAs. Limitations include (1) a lack of dietary data, as fibre and protein intake influence SCFAs, (2) the inability to assess other COPD phenotypes, such as chronic bronchitis and rapidly progressing COPD, (3) the cross-sectional design, which limits causal inference. Consequently, our data cannot infer the directionality of the associations, which may result from SFCAs’ effect on COPD disease, or secondary to COPD-related changes in activity, nutritional intake, or frailty-related microbiome changes, rather than being primary contributors to disease development. (4) multiple statistical testing across multiple SCFA measures and several COPD-related outcomes without formal correction for multiple testing, increasing the risk of chance findings — results should therefore be interpreted as exploratory and hypothesis-generating, and (5) SCFAs are microbiota-derived, and although we required a 14-day antibiotic washout, residual effects cannot be ruled out. However, most participants had a substantially interval since their last antibiotic exposure. Time since last antibiotic treatment was recorded in 194 of 233 participants and exceeded 12 weeks for most participants (89 participants with COPD (92%), 90 controls (93%)). (6) Lack of specific data on ongoing inflammation in the small airways, the primary site of inflammation in COPD, which is where any SCFA-related immunomodulatory effects in COPD are most likely to occur. Specific markers from immunohistology or CT (e.g., mucus plug scores, airway wall thickness) could enable further patient stratification and reveal associations not apparent in the current analysis.

## Conclusions

Plasma formate concentrations were consistently lower in patients with COPD compared with controls. Several SCFAs aligned with key COPD phenotypes, including FEV_1_ <50%, frequent exacerbations and symptom burden. Together, our findings outline a potential metabolic signature, while keeping the dual origin of formate in mind. Future studies should elucidate the mechanisms linking SCFAs to COPD, explicitly including the often-overlooked formate, and employ longitudinal and interventional designs to test therapeutic potential.

## Data Availability

The datasets generated and/or analysed during the current study are available in the Dryad repository, [10.5061/dryad.mw6m9069h].

## Abbreviations

CAT: COPD Assessment Test
CI: Confidence interval
COPD: Chronic obstructive pulmonary disease
CT thorax: Thoracic computed tomography
EBC: Exhaled breath condensate
FEV_1_: Forced expiratory volume in 1 s
FVC: Forced vital capacity
GC-MS/MS: Gas chromatography–tandem mass spectrometry
GLS: Generalised least squares
HU: Hounsfield units
LAA: Low attenuating area
NMR: Nuclear magnetic resonance
OR: Odds ratio
SCFA: Short–chain fatty acid
SD: Standard deviation
s%: Sympercent
